# Symbolic Convergence or Divergence? Making Sense of (the Rhetorical) Senses of a University-Wide Organizational Change

**DOI:** 10.3389/fpsyg.2021.690757

**Published:** 2021-07-28

**Authors:** Lina Ba, W. G. Will Zhao

**Affiliations:** ^1^Wuhan Technology and Business University, Wuhan, China; ^2^School of Management, University of Bath, Bath, United Kingdom; ^3^Faculty of Business Administration, Lakehead University, Thunder Bay, ON, Canada

**Keywords:** organizational change, fantasy theme analysis, rhetoric, organization members, change sensemaking

## Abstract

This research investigates the extent to which organizational change initiatives may lead to divergent patterns of sensemaking among organizational members. Drawing on the symbolic convergence theory, we performed an in-depth fantasy theme analysis of organization members’ rhetoric around an organizational change at a private university. Our analysis uncovers six fantasy themes and two corresponding fantasy types, which lead to no rhetorical vision. The lack of cognitive convergence between change initiators and change recipients suggests the inherent incompatibility between managerial and employee fantasies around organizational change, barring the exceptions of dual-responsibility change recipients (e.g., faculty members who also assume administrative responsibilities), who tend to adopt the change initiator rhetoric. Overall, this study informs our extant knowledge of change sensemaking with novel theoretical and methodological insights and bears implications for organizational change researchers and practitioners alike.

## Introduction

Organizational change has become one of the most prominent topics in organizational research over the past decades (Weick and Quinn, 1999), with much research conducted within the framework of sensemaking (Weber and Manning, 2001; Chaudhry et al., 2009; De Vos and Freese, 2011). However, extant research has rarely approached organizational change sensemaking from rhetorical perspectives, despite the fact that rhetoric, or the purposive use of language to generate meaning (Hoffman and Ford, 2010; Zhao, 2017), has been regarded as an important mechanism to construct and reconstruct organizational facts (Alvesson, 1993) and to illuminate the emergence or obstruction of collective meaning in organizational life (Finstad, 1998).

To fill this important gap in our knowledge, this paper draws on the symbolic convergence theory (SCT; Bormann, 1972, 1982) in qualitatively investigating the sensemaking of different organizational members implicated in organizational change, with the research objective to reveal the potential convergence and/or divergence of sensemaking by different members of a rhetorical community (Olufowote, 2017). Empirically, we conduct an in-depth fantasy theme analysis (FTA) of organization members’ rhetoric around a compensation scheme-related organizational change at a leading Chinese private university.

In recent years, to better compete in the higher education sector, many private universities have sought to enhance the attractiveness of the compensations they offer to faculty members. In the fall of 2018, a leading private university in central China developed a new compensation scheme with collaborations from a consulting firm, which included performance bonuses that incentivize instructors to serve a longer term at the university and to conduct research in addition to teaching. However, while this new scheme was designed to boost staff morale, the contrary was observed, e.g., many instructors calling in sick, showing up late for class, taking annual leave in the middle of the semester, or start looking for new jobs elsewhere.

Interested in understanding the sensemaking aspect of the organizational change initiative that has potentially become ineffective, we delve into the sensemaking of different organizational members. To go beyond existing knowledge, we included in our scope both change recipients (Bartunek et al., 2006) and change initiators. Specifically, we explore the following two interrelated research questions:

*RQ1: What does organizational members’ rhetoric tell us about their collective sensemaking of an organizational change?*

*RQ2: To what extent do change recipients and change initiators converge in their sensemaking?*

We collected interview data with ordinary faculty members, chairs, managers, and other administrators, texts from organizational members’ *“reflective journals”* which existed prior to this study (see “Methods and Data” for information on these journals), and other public data on this organizational change. Specifically, we examine the setting, character, and action “variables” in said rhetoric which form the basis of fantasy themes and evaluate the prospects of fantasy themes converging into fantasy types, and fantasy types into rhetoric vision, respectively.

Our fantasy theme analysis (FTA) of the organizational members’ change-related rhetoric reveals that change initiators and change recipients are deeply divided on how to define the nature and implications of the organizational change, with exceptions of dual-responsibility change recipients (e.g., faculty members that assume administrative responsibilities), who tend to adopt the change initiator rhetoric. Managerial sensemaking chiefly revolves around the prospect of enhanced organizational support. Meanwhile, the rhetoric of average instructors suggests that the change negatively impacts their career and their work-life balance. The stark contrast between two collective patterns of sensemaking points to the undermined legitimacy of the organizational change initiative.

Overall, this research adds, in a few meaningful ways, to ongoing efforts at the intersection of organization theory and sociopsychology to treat sensemaking as a central social process in organizational change. For starters, drawing on the symbolic convergence theory (SCT; Bormann, 1972, 1982), this research furthers the theoretical development on change sensemaking by focusing on the convergence or divergence prospect of organizational members’ symbolic worlds. At the same time, our deployment of the FTA also makes a methodological contribution by demonstrating how alternative structural interpretations of sensemaking can be made beyond conventional textual analysis (Bartunek et al., 2006), i.e., with the fantasy theme, fantasy type, and rhetorical vision as building blocks (Park et al., 2016) of collective sensemaking.

Second, the sensemaking literature has been criticized for underexploring and undertheorizing the process of communication divergence (Dougherty et al., 2009) and favoring a sided account on the employees (Bartunek et al., 2006) or the managers (Luscher and Lewis, 2008). Our paper explores and juxtaposes collective sensemaking rhetoric by both change initiators and change recipients as change initiatives go forward, revealing the different patterns of sensemaking around a common symbolic object (i.e., organizational policy change in our case) within an organization.

Third, our empirical analysis adds to our existing knowledge of FTA. Joining existing research that challenges the convergence ideology of the SCT (Dougherty et al., 2009), we incorporated an element of conflict in our analysis (Olufowote, 2006), allowing the taken-for-grantedness of cognitive convergence for well-meaning organizational change initiatives to be further challenged; i.e., instead of taking the formation of rhetorical vision for granted, this paper documents a scenario of organization-wide rhetorical vision being unachieved and, thereby, extends the application of the FTA to the unrealizable.

The remainder of the paper is organized as follows: The second section revisits our extant knowledge on sensemaking and organizational change, and introduces the SCT and FTA. We then discuss the research design and overview the background of the organizational change, before delving into an in-depth FTA. This paper concludes with some remarks on some of the possible contributions this research could make to theory and practice.

## Theoretical Orientations

### Organizational Change and Sensemaking

To cope with various pressures for its survival and success, contemporary organizations try or are forced to introduce changes to their way of work at an ever-accelerating pace (Ala-Laurinaho et al., 2017). Much research on organizational change has pointed to the surprisingly high level of failure or less-than-desirable results (Kotter and Schlesinger, 1979; Awad et al., 2013). Many common approaches to organizational change end up causing resistance (Pardo Del Val and Martínez Fuentes, 2003) from employees, who may experience considerable stress from the organizational change in their daily work (Stouten et al., 2018). As the success of organizational change has been argued as depending largely on the employees (Shah et al., 2017), researchers have stressed the need to sufficiently address the people side of change (Arazmjoo and Rahmanseresht, 2020).

One prominent perspective for unpacking the people aspect of organizational change is sensemaking (Luscher and Lewis, 2008; Maitlis and Christianson, 2014; Helpap and Bekmeier-Feuerhahn, 2016). Over the last few decades since its initial introduction (Louis, 1980), sensemaking has been championed by organizational scholars as a central construct for studying change (Weick, 1995; Gioia and Thomas, 1996; Weick et al., 2005), particularly in explaining and describing cognitive processes in response to change (Helpap and Bekmeier-Feuerhahn, 2016). Sensemaking pertains to a process of assigning meaning to experiences (Kramer, 2016) and entails constant redrafting of emerging, credible stories (Weick et al., 2005). Faced with new experiences, or moments of ambiguity or uncertainty, organizational members try to make sense of a situation by picking up and interpreting cues from their environment, in a bid to arrive at a plausible explanation for what has happened (Weick, 1995).

Organizational change and sensemaking have intricate relations. On the one hand, it is particularly critical for maintaining a coherent understanding of organizational realities in a context of turbulence and change (Ala-Laurinaho et al., 2017). Organizational change is often full of ambiguous moments that force members of the organization to engage in sensemaking so that changes become meaningful (Maitlis and Sonenshein, 2010; Maitlis and Christianson, 2014). For instance, during the period of change, the cognitive shift of employees (Helpap and Bekmeier-Feuerhahn, 2016) or other change recipients (Bartunek et al., 2006) may trigger changes in their day-to-day patterns of behavior (Maitlis and Sonenshein, 2010). On the other hand, the existence of sensemaking implies that organizational change is more than a simple, linear process of implementing a macro-level initiative. Indeed, organizational change often unfolds in an unexpected manner (Balogun and Johnson, 2005), as individuals and groups participate in the reconstruction of meaning in the world around them with different cognitive schemata.

However, while Weick’s theory leaves room for divergence in organizational members’ sensemaking, it does not offer much insight into the extent to which divergence occurs (Dougherty et al., 2009). Following more recent insights of sensemaking (Persson, 2013), we argue that any researcher seeking a fuller appreciation of organizational change sensemaking should remain open about the possibility of divergence or non-convergence in organizational members’ sensemaking. After all, although sensemaking seems to be an individual experience, it is also a social intersubjective process (Weick, 1995).

### SCT and FTA: Bringing Conflict Back in

One important way to approach organizational change is to regard it as a rhetorical process (Finstad, 1998). Rhetoric explains the ways in which organizations attempt to achieve specific goals (Zhao, 2017) and helps understand how our social world is socially constructed (Ihlen and Heath, 2018). Fine (1996: 91) regards rhetoric as “repertoires of meanings” that the organizational members use to represent their work. Researchers also observed that the repertoires of images underlying different rhetoric might not be entirely consistent, thus revealing tensions among organizational members (Kitay and Wright, 2007). In other words, organizational change may be a distributed imagining process (Weick, 2005) where meaning sharing is not taken for granted (Peng, 2018).

We concur with these insights, yet, while we believe analyzing rhetoric would be a fruitful direction to understanding the sensemaking of organizational change, we also believe that we need tools that can structurally show the potential divergence and/or convergence of senses made by different organizational members, as “there is no reason to assume recipients and change agents share the same understandings” (Bartunek et al., 2006: 183). For such tools, we now turn our attention to FTA and the SCT underlying this approach.

Bormann (1972) developed the SCT drawing on the work of social psychologist Bales and his colleagues on group interactions (Bales, 1970). In studying group communications, Bales noticed the phenomenon of “dramatization,” i.e., when interactions go well, verbal and non-verbal communications become interlinked, which indicates that group members are involved in a common drama. Bormann (1985) extended this view to larger-scale group interactions in his SCT, which argues that individuals’ personal symbolic worlds could converge as group communications provide a basis for each other to form groups, discuss common experiences, and reach mutual understanding.

The SCT can help us understand whether and how cognitive and communicative cohesion emerge in organizations (Olufowote, 2017; Hossain et al., 2019). If a set of symbols for an event is passed from one individual to another, and both individuals shared said symbol about the event, then the symbol can spread to other individuals. If accepted, a collective reality is constructed for the group. Through studying these symbols rhetorically, it is possible to determine whether there are the same values and beliefs that bind a group together (Olufowote, 2017).

The SCT as the theoretical framework has been coupled with the empirical framework FTA, which entails understanding fantasy, its themes, types, and the rhetorical vision attributable from rhetorical narratives. Fantasy is a creative and imaginative interpretation of events, and it fulfills individuals’ psychological needs to label the past, the present, and the future (Bormann, 1982). This definition of fantasy is akin to the “senses” in the sensemaking literature, which carries the ascribed meanings, emotional response, and perceptions of the impacts of events (Bartunek et al., 2006). One advantage of analyzing sensemaking in light of the SCT is that the theory has built-in tools to not only account for the content (i.e., rhetoric) of sensemaking, but also assess the convergence prospect of these senses made (Hossain et al., 2019). Specifically, this is achieved through a systematic treatment of different building blocks of a collective symbolic process, i.e., fantasy themes, fantasy types, and rhetorical vision (Park et al., 2016).

A fantasy theme has three elements: The setting theme, the character theme, and the action theme (Bormann, 1972). Setting themes describe where actions occur; character themes revolve around agents’ roles and motivations; action themes refer to the plot clues from which actors act. The fantasy type, as a higher-level construct, is formed when a series of similar scenes, characters, and action themes are shared by members, which have the potential to become a framework of reference (Park et al., 2016). Similarly, when many fantasy types are gradually merged into several recurring dramatic plots, a rhetorical vision is formed, which then becomes part of a common symbolic reality that impacts the participants of an interaction. With FTA, it is possible to explore in detail organizational members’ rhetoric and understand in a fine-grained manner whether fantasies by some organizational members initiating the change may appear reasonable to other members, and whether cognitive convergence takes place.

Despite their theoretical and empirical value, SCT and FTA have been criticized by some research as suffering from a convergence ideology where the symbolic process is thought to be conflict-free (Olufowote, 2006; Dougherty et al., 2009), especially when it comes to the generation of rhetorical vision. Treating the sharing of personal consciousness and meanings as a “warranted occurrence” limits SCT’s potential and creates blind spots that obscure other communication processes (Olufowote, 2006). We concur with this critical reading of SCT against obscuring the fundamental ideological conflicts or overemphasizing the creation of shared meanings.^1^ With this conflict vigilance, we now empirically explore organizational change sensemaking.

## Methods and Data

### Contextualizing the Organizational Change

To become more competitive in the higher education sector, to increase faculty members’ morale, and to attract more talents to join the university, the focal university in this study spent more than 6 months designing a new compensation scheme with the help of a consulting firm, which sent a team to work on campus for about 2 months. The team conducted a series of interviews with faculty members and administrators to gain a better understanding of what kind of schemes for faculty members would best achieve the university’s goals.

At the end of the two-month period, they recommended investing a confidential amount of money to fund an overall salary increase and introduced a complex bonus scheme involving three criteria, namely, tenure at the university, seniority of the position held, and research excellence. This new change proposal was accepted and announced by the university, and the administrators began to initiate and facilitate the change. In the 8 weeks immediately following the announcement of the change, the consulting firm asked members of the faculty and management to keep a “reflective journal” where they can write their thoughts about the new scheme (such journals will serve to triangulate with our interviews which were conducted separately from the consulting work – see more details below).

### Data Source

Interested in understanding the organizational change initiative’s psychological aspect that has potentially become ineffective, we delve into different organizational members’ sensemaking. To go beyond existing knowledge, we included in our scope both change recipients (Bartunek et al., 2006) and change initiators. Specifically, we sought to interview organizational members in each professional category, including faculty members (lecturers, professors, and chairs) and administrators. Initially, we identified 23 potential informants through the university’s Web site and contacted them through email or phone (when such information was available). When selecting the informants for our study, we used a comprehensive set of criteria for both the faculty members and the administrators; i.e., the informants should (1) have performed actual teaching or administrative duties for more than 1 year, (2) be aware of the organizational change, and (3) have submitted at least one reflective journal entry (originally submitted to the consulting firm responsible for the new scheme). We explained to the informants that they would participate in the study voluntarily and could withdraw at any phase of this study, and that our interview was to give them a voice about the organizational change while maintaining their anonymity.

The final list of informants included 13 individuals that fully met our selection criteria and that consented to participate in the interviews. The interviews were face-to-face, unstructured, and typically lasted 30–90 min. During the interviews, the first author would begin with an open-ended question, “Tell me how you feel about your work with this new scheme in place?” Additional notes of the facial expressions and tones were taken during the interviews. Questions such as “how does/did that make you feel?” were constantly used to assess informants’ feelings. Table 1 summarizes their demographics and their positions in the organization.

**Table 1:** Informants’ demographics and positions.

Informants	Position	Role in change	Age	Gender	Tenure
P1	Lecturer	Recipient	34	F	3
P2	Lecturer	Recipient	37	F	5
P3	Lecturer	Recipient	35	M	3
P4	Lecturer	Recipient	30	F	2
P5	Lecturer	Recipient	35	M	4
P6	Full Professor	Recipient	48	M	12
P7	Chair of Department (Associate Professor)	Recipient	42	M	8
P8	Discipline Head (Associate Professor)	Recipient	39	F	7
P9	Chair of Department (Associate Professor)	Recipient	50	F	10
P10	Director of Human Resources	Initiator	45	F	8
P11	Director of Administration	Initiator	39	F	5
P12	Associate Director of Finance	Initiator	49	M	7
P13	Head of Development and Planning Office	Initiator	41	F	5

Insights that emerged from the interviews were later triangulated with (1) reflective journal entries submitted by the informants to the afore-mentioned consulting firm (both the firm and the informants gave us consent for collecting these entries) and (2) publicly available data (e.g., from the university’s Web site) on the organization’s past and current compensation policies, consulting firm’s reports, and other communications by the university related to the change initiative.

### Analytical Procedure

After collecting the above data, the first author, together with a graduate research assistant, transcribed the interviews from records. The second author verified the transcripts against the records and re-transcribed the portions of the interviews when a gap of information was identified. After having had initial sensing of the interview data, we decided that the interview data and the journal entries were consistent in the topics covered and that the interviews did not contain fewer insights than the journal entries and therefore could be relied on as primary data sources for coding.

We then conducted a detailed FTA following the coding method of Park et al. (2016). First, we extracted the content of the informants’ rhetoric on organizational change. Fantasy themes, as the basic unit of analysis of fantasies, were identified when we came across the creative and imaginative interpretations of events (Bormann, 1982) that involve the three basic elements, i.e., setting, character, and action. Table 2 provides an overview of the fantasy themes, including definitions of the setting, character, and action themes, their scope in our setting, and how they were generally referred to in the data.

**Table 2:** Coding fantasy themes.

	Definition	Scope	Reference
Setting	The place where actors develop their actions	Broadly, the organization, which presents a natural setting of actors’ day-to-day interactions and performing their duties, and family, where non-professional activities occur	University, departments, offices, and home
Character	Main actors involved in the narratives	All actors having a stake in the organization’s activities are considered, with special foci on their roles in and outside professional life	Teaching staff, administration, and faculty with administration responsibilities
Action	Behaviors embedded in the narratives	Actions that are pertinent to fulfilling ones’ professional activities, e.g., teaching and research, and family duties	Working at school and leading personal lives

Then, we categorized these themes into fantasy types when possible. The classification of fantasy themes into fantasy types is the process of assigning a shorter label for fantasy themes that are alike, which, when aggregated, potentially take a larger place in the ultimate rhetorical vision in which many people participate. Quantitatively, this meant to decide a fantasy type when at least two related fantasy themes could be identified as converging (Bormann, 1972, 1982).

The categorization was subject to constant revision, with new categories being created and others modified (Florek et al., 2006). The emergence and modification of these categories required a review of previously analyzed data to apply the same categorization criteria across the dataset. After the fantasy types emerged, the last step of FTA was to examine the convergence prospects of fantasy types and to construct the rhetorical vision, which embodies the shared consciousness of communicative participants and the frame of interpreting experiences (Kuypers, 2009). To reduce subjectivity, we discussed and debated the (non)convergence prospects of our categories until a consensus could be reached.

## Results

Our analysis of various organizational members’ change rhetoric revealed six fantasy themes, which were built into two fantasy types. These fantasy themes include (1) university is a rewarding workplace, (2) university is a workplace without proper support, (3) prioritizing research helps faculty members to thrive, (4) prioritizing research brings stress to faculty members, (5) change enhances work relationships, and (6) change encroaches family space. The fantasy types are (1) the new scheme will bring more opportunities for employees, and (2) the new scheme will create more challenges for most employees. No organization-wide rhetorical vision was found across members of different positions. Interestingly, though managerial and faculty members’ fantasies around the organizational change were inherently incompatible, we found that for different groups of organizational members, i.e., change initiators and change recipients, there was a strong intra-group consensus in terms of how members understood the organizational change. Indeed, at the faculty level, the new compensation scheme seems to have created a general sense of stress and urgency, largely due to its pertinence to the changed scope of work. On the management side, organizational change is understood as paving the way for improving employees’ productivity by enhancing their income.

In other words, barring the exceptions of dual-responsibility change recipients (e.g., faculty members that also assume administrative responsibilities), who tended to adopt the change initiator rhetoric, what we observed was a stark contrast in sensemaking between two groups of a rhetorical community. This made us believe that while there was no rhetorical vision on the organizational façade, beneath the lack of rhetorical vision may be two competing rhetorical visions^2^ that each existed for a sub-organizational rhetorical community (Endres, 1994; Olufowote, 2006). Indeed, change initiators seemed to have adopted a rhetorical vision that prioritized research and performance; meanwhile, change recipients adopted a rhetorical vision that prioritized organizational support. Table 3 summarizes the results of our analysis.

**Table 3:** Fantasy themes, fantasy types, and rhetorical vision.

Fantasy themes (*N* = 6)	Fantasy types (*N* = 2)	Informants’ positions	Change initiator or recipient	Competing (sub-organizational) rhetorical visions	Organization-wide rhetorical vision
University is a rewarding workplace.Prioritizing research helps faculty members to thrive. Change enhances work relationships.	The new scheme will bring more opportunities for employees.	Managers and administrators	Initiator	Change facilitates scholarly performance.	The rhetorical revision was non-existent as the sensemaking patterns by different groups in the rhetorical community were in stark contrast with each other.
		Professors with managerial duties	Recipient		
University is a workplace without proper support. Prioritizing research brings stress to faculty members.Change encroaches family space.	The new scheme will create more challenges for most employees.	Regular instructors	Recipient	Change necessities organizational support.	

### Fantasy Themes and Exemplary Quotes

Organizational members’ sensemaking around the nature of the university differs greatly. For managers, the change initiative caters to the research orientation of many universities these days and is especially attractive to instructors. The average instructor, however, thinks the change will invade their family lives. Below are the fantasy themes on the nature of the change and some exemplary quotes.

#### University Is a Rewarding Workplace

Managers generally portray the university as an ideal workplace where hard work will be rewarded, as evidenced by the following excerpts,

“Rising incomes help instructors find the dignity of their profession and begin to do a better job at educating people… We want the new hires to be absorbed by our culture of ‘get paid for your input.’ Research is supported, publications are rewarded, and new hires will have a sense of belonging and recognition” (P10).

“At our university, you will never have to worry about if you have the right platform for your skills. Your talents can be fully displayed” (P11).

“We had policy changes before, and you know that some of the policies were like propaganda… They are exaggerated. But after a careful reading of this compensation policy, I found that this one is real” (P13).

#### University Is a Workplace Without Proper Support

For the instructors, the new scheme does not seem to address their concerns about the right kind of support they need. Our informants typically mention the time and guidance as to two things they need most as support.

“I hope to have more time to learn, and also I have a multi-disciplinary position, I need more opportunities to learn different knowledge, I need to go abroad to broaden my horizons, but how can I arrange the work with the department?” (P1).

“Sometimes I do not know if I’ve done a good job on my research. I am sure actually I have not done a good job. I want to be guided and checked by an experienced professor” (P3).

“Services at all levels of the school should be better integrated when it comes to research support” (P4).

By contrast, administrators tend to think that help is available for the instructors who need to adapt their work rhythm. For example, the following quote shows a clear “I am here to help” attitude of the administrators.

“There are a lot of forms. Instructors feel the work hours are long, and the pressure is higher, so I take time to guide, communicate with, and help instructors, which brings my work ability to a higher level, to make the impossible possible and to solve the problem step by step” (P12).

#### Prioritizing Research Helps Faculty Members to Thrive

To the administrators, a university career is especially ideal for individuals who want to develop their research talents to the full. The change initiative will make that possible. Here is an example:

“After the new policy (is in place), everyone can play at their advantages. The incentive mechanisms fit very well with the values of freedom and individuality that many instructors have” (P12).

Noteworthily, the university’s emphasis on research tends to be viewed in an extremely positive light by professors who also hold administrative responsibilities, such as the chair. To many of them, the change is understood to give instructors enough incentive to do better research and to transition toward balanced teaching and research.

“Instructors are under a lot of pressure at work for sure, so we have to be patient and then lead by example. In the end, instructors will turn the pressure into motivation and get over it” (P7).

“An instructor is not just a disseminator of knowledge for a student in class but has the opportunity to be a producer of knowledge…. this new policy is a real drive. From finding data, literature reviews, data analysis, to working with editors and reviewers, I can share my experience. It’s much easier for young people nowadays” (P8).

“Research is linked to promotion. You should be busy but happy. Time management. By investing in research, we can build a better career. Do not forget, research is a job where you have control over your own working hours” (P9).

When asked about her role in facilitating research, the same chair remarked that

“For research, my job is mainly to let instructors learn in practice, and help them in ways I can, such as grouping instructors together, which makes research much easier” (P9).

#### Prioritizing Research Brings Stress to Faculty Members

While a more junior faculty member (P4, a recent PhD graduate) also voices support for such a change, average instructors typically portray the university’s emphasis on research as a cause for great stress. Many instructors point out that the research requirement means that they were forced to increase the workload, adapt the workflow, and change their attitude to colleagues who are good at research (e.g., P1, P3, P5, and P6).

“I was new to the job and was worried about low pay, but it seemed like the new policy was very friendly to young PhDs like us. With research rewards, I am confident, and now the whole department knows that I can write papers” (P4).

“After the new policy, we are given a lot of options, and there is a feeling [pause] that it has something to do with one’s ability to work. There is pressure” (P1).

“There is a lot of pressure at the moment, especially since the new policy was created, and the whole research model and concept has changed a lot. For example, it would require research in English, but I did not study it systematically, so it’s a bit hard to change” (P3).

“To be honest, with the new policy, some colleagues feel stressed; they need some data for research, but they do not know how to get it. There was no previous requirement for such work” (P5).

“After 12 years (of working here), I really feel that this new policy - to be honest with you - will make me look bad among young colleagues. I need to change my attitude to them” (P6).

#### Change Enhances Work Relationships

Administrators and professors with dual responsibilities generally describe the change initiative as a way to foster stronger team spirit among instructors, as illustrated by the following examples:

“This is why we have changed our policy to encourage teams to do research, which makes it easier. Instructors can meet more like-minded people in their research groups than their own departments” (P7).

“So we encourage creating new research groups, because that atmosphere of the team will be what instructors wanted” (P11).

While this view is echoed by some instructors who feel that the establishment of research groups will give them extra exposure to good work relationships (e.g., P5), most instructors are discontent about the imposed social interactions with their colleagues after working hours.

“Everyone needs a group. I like the kind of partnership where we can grow together and help each other” (P5).

“Sometimes, I still plan and arrange things for the next day when I go home. I also receive work-related WeChat *(an instant messenger)* notifications very late at night. I am at home, and my heart is at work” (P2).

“I often work late and sometimes have meetings on weekends. It is difficult to balance work and family. They are irreconcilable in some respects” (P5).

#### Change Encroaches Family Space

For many instructors, the change overburdens them, as they need to expend a lot of energy at work, which will make them emotionally exhausted or too tired to fulfill their family obligations.

“My son is in junior high school. His father and I are busy working and do not have much time to spend with him. I need to adjust myself and talk to him more often when I have time” (P2).

“Sometimes when I’m too busy to take care of my family, my family is still understanding, and I feel guilty, especially for my children, my energy is limited” (P3).

“There will be an increased conflict between life and work. I am already work-oriented. Sometimes I promised my child something, but then suddenly I cannot go, I feel very guilty” (P5).

The above analysis of the elements of the fantasy themes suggests a deep divide between management and the average faculty members, which prompts us to re-appreciate organizational change as distributed imagining processes (Weick, 2005; Peng, 2018). Contrary to mainstream change sensemaking studies, the lack of rhetorical vision for the organization highlights the necessity of separating managers (Luscher and Lewis, 2008) and employees (Helpap and Bekmeier-Feuerhahn, 2016) *or change initiators and change recipients* (Bartunek et al., 2006) when analyzing and evaluating organizational change.

From the change initiators’ perspective, in this case, organizational change is an occasion for employees to receive more support from the organization. Possibly motivated by external pressure for raising its research profile, the university worked to give instructors incentives to do more research. Administrators regarded this work as creating a research-friendly atmosphere that incentivizes the employees to make research a part of their career. Unsurprisingly, in this sensemaking, the new initiative will bring out positive change; the relationship between superior and subordinate is improved, and the sense of team is being forged. In such an atmosphere, the employee’s satisfaction will be high; the enterprise will naturally retain the employees, but also attract more talents to join the organization. For dual-responsibility change recipients (faculty members that also assume administrative responsibilities), organization change has been understood more in line with the managerial sensemaking, though they at times struggled to rationalize their roles (Luscher and Lewis, 2008).

By contrast, for the average employees or change recipients, in this case, organizational change, while potentially empowering some members, poses more challenges. Not having been presented with an immediate positive improvement brought by the new initiative, the employees viewed the change in a more negative light. Average faculty members expressed their need for more training and time to adapt to the new requirements entailed in the change. In their sensemaking, the university needs to give more support to compensate for the increased pace and enlarged scope of work required by the change, which renders work-family balance simply unachievable – all this cannot easily be compensated for with a higher salary or bonus.^3^

## Discussion and Conclusion

Interested in unpacking the sensemaking related to a university-wide organizational change initiative, this research performed an FTA on the change-related rhetoric of different organizational members, including the change initiators and change recipients. Our FTA reveals that change initiators and recipients are deeply divided on how to make sense of the organizational change. Managerial sensemaking chiefly revolves around the prospect of enhanced organizational performance. Meanwhile, average instructors’ rhetoric points to the negative impact of the change on their professional and family lives. The stark contrast between two collective patterns of sensemaking suggests the potential existence of competing rhetoric visions for different groups in a rhetorical community and the non-existence of an organization-wide rhetorical vision.

Overall, our study adds to the ongoing discussions of organizational change sensemaking in a few meaningful ways. First, with a careful SCT-based qualitative analysis of organizational members’ rhetoric, this research furthers theoretical development on change sensemaking. Meanwhile, this paper’s mobilization of the FTA also makes a methodological contribution by demonstrating how sensemaking and its convergence or divergence can be more structurally interpreted with the fantasy theme, type, and rhetorical vision as building blocks (Park et al., 2016). Specifically, our analysis allowed us to systematically showcase the extent to which rhetorical visions existed in a rhetorical community; i.e., in our case, there was no organization-wide rhetorical vision, yet competing sub-organizational rhetorical visions may have emerged for members of different positions within an organization. Equally important, our analysis reveals the nuances of organizational cognitive non-convergence or divergence (Levesque et al., 2001; Shams, 2019); e.g., dual-responsibility change recipients’ symbolic world seems to overlap with change initiators more than fellow change recipients.

Second, since the publication of Weick’s seminal work on sensemaking in organizations (Weick, 1995), the flourishing empirical work still often blackboxes collective sensemaking, leaving a gap for studying organizational change sensemaking between different groups of organizational members (Dougherty et al., 2009). Our research provides a more balanced account of different organizational members’ change sensemaking, rather than focusing on the change recipients’ (Bartunek et al., 2006) or administrators’ side (Luscher and Lewis, 2008). Specifically, our analysis focuses on the setting-actors-action elements of organizational members’ rhetoric makes explicit the similarities and differences of the symbolic worlds of members in a rhetorical community (Olufowote, 2006). The highly polarized rhetoric from both ends of the managerial-managed continuum reveals organizational members’ cognitive divergence, showcasing the non-existence of an organization-wide rhetorical vision. Equally noteworthy in our findings is the potential existence of sub-organizational rhetorical visions for the employees and the managers, respectively.

Connected to this, the SCT has been criticized for overemphasizing the emergence of shared meanings (Olufowote, 2006) and for trivializing communication divergence (Dougherty et al., 2009). Our systematic analysis and juxtaposition of change recipients’ and change initiators’ sensemaking highlight the conflicts within a rhetorical community, thus loosening SCT’s convergence ideology. By documenting the unachieved symbolic convergence (into an organization-wide rhetorical vision), the study extends the application of the FTA to organizational communicative processes otherwise overlooked in other FTAs (Park et al., 2016; Hossain et al., 2019), i.e., where the formation of rhetorical vision tends to be taken for granted.

This research also bears some managerial implications for the promotion of organizational change. As our research reveals, most employees in an organization share similar understandings as to whether increased pay and bonuses will compensate for the changing nature and the enlarged scope of their work. Our study is set in the higher education sector, where there has been increasing demands for faculty members to assume multiple responsibilities, i.e., teaching, research, and service. In order to better carry out these responsibilities, academics may need more training and more time for adapting to the new requirement regarding one or more of their responsibilities. This difference between change initiators and recipients in their understanding of the meaning of organizational change reminds organizational decision-makers of the sobering fact that organizational change, no matter how well-intended, is generally not well received. Organizational change practitioners, therefore, need to understand that while improvements in compensation can mitigate some dissatisfactions, much more needs to be done. Only when support is in place for all potentially affected organizational members’ can the likelihood of resistance be alleviated to the greatest extent.

Organizational change success cannot be taken for granted. By critically mobilizing the SCT framework and by demonstrating the potential of FTA as an analytical framework for exploring the cognitive process in organizational change, our research is of interest to the academic community and may provide the seed for further application of SCT and FTA in other similar organizational contexts. Nevertheless, this study has some limitations. First, while this study examined the change sensemaking of both change initiators and recipients, our data did not allow for an exploration of how sensemaking by different sides may change over time. More research is needed to analyze and compare the responses of managers and non-managing organizational members at different stages of organizational change. Second, our study is based on observations of a small number of organizational members at a private higher education institution and of a compensation-related organizational change. Future research can assess the (non-)convergence prospect of change sensemaking with a larger sample size and in different organizational change contexts, so as to examine the applicability and transferability of our findings.

## Data Availability Statement

The raw data supporting the conclusions of this article will be made available by the authors, without undue reservation.

## Ethics Statement

The studies involving human participants were reviewed and approved by Wuhan Technology and Business University Research Ethics Committee. Written informed consent for participation was not required for this study in accordance with the national legislation and the institutional requirements.

## Author Contributions

WZ and LB contributed to conception and design of the study and performed the analysis. LB collected the data. WZ wrote the first draft of the manuscript. All authors contributed to manuscript revision, read, and approved the submitted version.

## Conflict of Interest

The authors declare that the research was conducted in the absence of any commercial or financial relationships that could be construed as a potential conflict of interest.

## Publisher’s Note

All claims expressed in this article are solely those of the authors and do not necessarily represent those of their affiliated organizations, or those of the publisher, the editors and the reviewers. Any product that may be evaluated in this article, or claim that may be made by its manufacturer, is not guaranteed or endorsed by the publisher.

## Footnotes

^1^We are indebted to the anonymous reviewers for encouraging us to adopt a more critical reading of the SCT.

^2^We are indebted to one anonymous reviewer for the insight on the potential existence of competing rhetorical visions.

^3^We thank one anonymous reviewer for raising this excellent point.
